# Differing Patterns of Altered Slow-5 Oscillations in Healthy Aging and Ischemic Stroke

**DOI:** 10.3389/fnhum.2016.00156

**Published:** 2016-04-13

**Authors:** Christian La, Pouria Mossahebi, Veena A. Nair, Brittany M. Young, Julie Stamm, Rasmus Birn, Mary E. Meyerand, Vivek Prabhakaran

**Affiliations:** ^1^Neuroscience Training Program, University of Wisconsin–MadisonMadison, WI, USA; ^2^Department of Radiology, University of Wisconsin–MadisonMadison, WI, USA; ^3^Department of Medical Physics, University of Wisconsin–MadisonMadison, WI, USA; ^4^Department of Psychiatry, University of Wisconsin–MadisonMadison, WI, USA; ^5^Department of Bio-Medical Engineering, University of Wisconsin–MadisonMadison, WI, USA

**Keywords:** rs-fMRI, low-frequency oscillations, fALFF, slow-5 oscillations, aging, stroke

## Abstract

The ‘default-mode’ network (DMN) has been investigated in the presence of various disorders, such as Alzheimer’s disease and Autism spectrum disorders. More recently, this investigation has expanded to include patients with ischemic injury. Here, we characterized the effects of ischemic injury in terms of its spectral distribution of resting-state low-frequency oscillations and further investigated whether those specific disruptions were unique to the DMN, or rather more general, affecting the global cortical system. With 43 young healthy adults, 42 older healthy adults, 14 stroke patients in their early stage (<7 days after stroke onset), and 16 stroke patients in their later stage (between 1 to 6 months after stroke onset), this study showed that patterns of cortical system disruption may differ between healthy aging and following the event of an ischemic stroke. The stroke group in the later stage demonstrated a global reduction in the amplitude of the slow-5 oscillations (0.01–0.027 Hz) in the DMN as well as in the primary visual and sensorimotor networks, two ‘task-positive’ networks. In comparison to the young healthy group, the older healthy subjects presented a decrease in the amplitude of the slow-5 oscillations specific to the components of the DMN, while exhibiting an increase in oscillation power in the task-positive networks. These two processes of a decrease DMN and an increase in ‘task-positive’ slow-5 oscillations may potentially be related, with a deficit in DMN inhibition, leading to an elevation of oscillations in non-DMN systems. These findings also suggest that disruptions of the slow-5 oscillations in healthy aging may be more specific to the DMN while the disruptions of those oscillations following a stroke through remote (diaschisis) effects may be more widespread, highlighting a non-specificity of disruption on the DMN in stroke population. The mechanisms underlying those differing modes of network disruption need to be further explored to better inform our understanding of brain function in healthy individuals and following injury.

## Introduction

The default-mode network (DMN) is considered a central network of the cortical system. Primarily comprised of the precuneus/posterior cingulate cortex (pC/PCC), the medial prefrontal cortex (mPFC), and bilateral inferior parietal lobules (IPLs), this network consists of regions actively recruited during a state of *rest*, where no goal-directed behavior is required (Fransson, 2005; Fox and Raichle, 2007; Raichle, 2010). In healthy individuals, in addition to being highly active during the passive condition of *rest*, activity within the DMN is actively suppressed during goal-directed task performance facilitating the various goal-directed processes (Raichle et al., 2001; Fransson, 2005; Fox and Raichle, 2007). Disruption of DMN network activity/de-activity pattern contributes to the impairment of functional networks associated with a variety of behaviors observed in cognitively impaired populations (Grady et al., 2006, 2010; Persson et al., 2007; Lustig and Jantz, 2014), such as a decline in speed of processing, executive and/or memory functions. Investigation of this network has gained popularity in recent years, in particular for its utility in describing aging and a variety of neurological (e.g., Alzheimer’s and Parkinson’s disease) and psychiatric (such as Schizophrenia, Autism, and ADHD) disease states, where abnormal DMN activity has been consistently found (Greicius et al., 2004; Persson et al., 2007; Damoiseaux et al., 2008; Greicius, 2008; Broyd et al., 2009).

Resting-state fMRI or rs-fMRI is a rapidly evolving method allowing one to explore the intrinsic low-frequency fluctuations (LFOs) and the intrinsic connectivity networks (ICNs) of the brain (Beckmann et al., 2005; Damoiseaux et al., 2006; Fox and Raichle, 2007; Cole et al., 2010; Schölvinck et al., 2010; Patriat et al., 2013). Analysis of functional connectivity (Friston et al., 1993), a method to assess the temporal correlation of distant brain regions, can be used to investigate the functional organization of the brain without an overt task or external input (Biswal et al., 1995; Van Den Heuvel and Pol, 2010). In addition, spatial patterns of resting functional activity can be extracted by computing the amplitude of the low-frequency fluctuation (ALFF; Yu-Feng et al., 2007). Because of the passive nature of the resting-state condition, a rs-fMRI scan is highly advantageous as it allows the investigation of patients that would otherwise have difficulty with task performance. This approach is less susceptible to variability in task-related behavior such as motivation and attention.

Recent studies using rs-fMRI have also demonstrated significant differences in the DMN in patients following the onset of a stroke, with lesion in regions not belonging to the DMN (Tuladhar et al., 2013; Park et al., 2014). Specifically, stroke patients exhibited decreased network co-activation within the regions of the DMN, primarily over the regions of the PCC. However, ischemic stroke is dissimilar to the aforementioned disease states (e.g., Alzheimer’s disease, Parkinson’s disease) in the acute nature of the injury, imposing rapid network changes and network re-organization, and thus may have a different mechanism of network disruption in comparison to more progressive disorders. Despite the disruption in vasculature, the investigations of stroke patients can bring new insight into the source and underlying mechanism of cortical network disruption. The investigation of stroke population also allows for an assessment of time-dependent, stroke-related cortical changes and cortical re-organization following initial onset, and permits the eventual longitudinal assessment of network recovery in the later stages of stroke.

Here, we aimed to investigate the disruption of the DMN—a network shown to be subjected to the diaschisis effect of the stroke lesion—and two selected task-positive networks occurring in patients following the event an ischemic stroke in a cross-sectional study using rs-fMRI. Specifically, we assessed these changes via an investigation of the distribution of LFOs in the frequency domain. The DMN is not regularly investigated in stroke population because of its low susceptibility to direct stroke-related lesion injury. However, the DMN has been demonstrated to be susceptible to changes through indirect mechanism such as diaschisis effects (Tuladhar et al., 2013; Park et al., 2014). Detailed investigations of amplitude information of those LFOs power spectra have been implemented by subdividing the frequency distribution of these spontaneous oscillations into distinct infra-slow frequency ranges (i.e., slow-5: 0.01–0.027 Hz, slow-4: 0.027–0.073 Hz, slow-3: 0.073–0.198 Hz, slow-2: 0.198–0.25 Hz; Penttonen and Buzsáki, 2003; Buzsáki and Draguhn, 2004; Zuo et al., 2010), with significant slow-4 and slow-5 oscillations demonstrated to be primarily restricted to gray matter; while slow-2 and slow-3 oscillations restricted to white matter (Zuo et al., 2010). Many areas exhibiting maximal low-frequency oscillation amplitudes were also found in regions of the DMN.

Using an approach of component fractional ALFF (fALFF), where estimates of relative spectral power are computed for network component oscillation (Calhoun et al., 2011; Gohel and Biswal, 2014), our group has previously provided evidence that implicated specific fluctuation within the slow-5 oscillations range (0.01–0.027 Hz) in the disruption of the DMN of stroke population in their later stage, unsettling the balance between slow-4 and slow-5 oscillation within the resting state, potentially disrupting the communication between distal nodes within a system [La et al., 2014; La et al., submitted]. This finding was in accord with the results from Zhu et al. (2015), where they found that regions with altered activity after stroke were more extensive within the slow-5 band. However, whether this reduction of oscillation power is unique to the DMN, or whether those changes extends beyond the DMN following the event of an ischemic stroke had yet to be explored and was investigated here. In this study, we examined the amplitudes of the slow-5 oscillations in three independent subcomponents of the DMN, as well as two components of ‘task-positive’ systems (primary visual and sensorimotor) for the investigation of stroke-related diaschisis effect on various network of the cortical system.

## Materials and Methods

### Participants

This study was designed as a cross-sectional rs-fMRI study to investigate aging and stroke related effects on the amplitude of resting state LFOs from selected network to better characterize the mechanism of network disruption. One hundred and thirty-seven participants were recruited in the study, with each participant invited to complete a single scanning session, which included a 10-min resting state fMRI scan and a high resolution structural scan (parameters described in the next section). Participants were recruited through the UW Hospital and Clinics, UW-Madison campus and city of Madison community. Participants in this study expressed no history or signs of neurological/psychiatric disorders. Other exclusion criteria included contra-indications to MRI, claustrophobia or pregnancy, and intake of certain types of medications (e.g., antipsychotics, antidepressants, sedative hypnotics, etc.). Participant presented no sign of compromised capacity or ability to consent, as established by neurological examination. This study was approved by the University of Wisconsin–Madison’s Health Sciences Institutional Review Board (IRB), and written informed consent was obtained from each of the participants.

Sessions presenting excessive motion (greater than 2-mm in each of the directions in *x*-*y*-*z* and roll–pitch–yaw), or presenting gross artifacts (ghosting, various erroneous susceptibilities, calcification artifacts, etc.) were removed from analysis. Subjects with lesion in the regions of the DMN were also excluded. A total of 115 individual resting-state fMRI sessions from the participants were further assessed. The participants were separated into four population categories: (1) stroke patients in their early stage (‘stroke-early,’ ≤7 days post stroke onset, *n* = 14, six non-cortical strokes), (2) stroke patients in their later stage (‘stroke-late,’ between 1 to 6 months post stroke onset, *n* = 16, six non-cortical strokes), (3) young healthy adult (YHA) subjects (*n* = 43), and (4) old healthy adult (OHA) subjects (*n* = 42). Patient information and demographics are provided in **Table 1**. More information regarding clinical, demographic, and session information for the 30 enrolled ischemic stroke patients can be found in Supplementary Material A. No subject was repeated between the time-points.

**Table 1:** Subjects demographic table.

	Sample size (*N*)	Gender	Mean age	Days after stroke onset median (range)	NIH-SS
Young healthy adults (YHA)	43	23M:20F	22.5 ± 2.7	n/a	n/a
Older healthy adults (OHA)	42	22M:20F	61.2 ± 7.8	n/a	n/a
Stroke patients in early stage (≤7 days)	14	11M:3F	65.0 ± 15.7	4.6 ± 1.9 4 (1–7)	3.4 ± 5.0 2 (1–15)
Stroke patients in later stage (1–6 months)	16	8M:8F	61.0 ± 10.4	110 ± 56.3 125 (24–180)	2.0 ± 2.8 1.5 (0–10)

*Gender differences in the recruitment of stroke-early subjects were non-significant (chi-square test, p = 0.61).*

In this study and analysis, Subject enrollment favored inclusion of healthy individuals to reduce the influence of specific lesions from our stroke population in the isolation of the independent components by ICA (i.e., ~40 subjects in each of the healthy control groups, and ~15 subjects in each stroke groups). The inclusion of an YHA group permitted the distinction between an age-effect and a stroke-effect. The difference in age between the young group and each of the other groups (OHA, stroke-early, and stroke-late patients) was significant (*t*-test, *p* < 0.05). No significant difference in age was found between the old, stroke-early, and stroke-late stroke groups, allowing us to control for age as a factor in our investigation of stroke-related brain changes. The inclusion of stroke-early and stroke-late subjects allowed for an assessment of the evolution of a stroke-related effect on remote networks including the DMN.

Head motion was assessed using a Euclidean norm (enorm) approach to characterize the possible contribution of head motion to the observed effects. Age was found to be a contributor to head motion during the resting-state scan, with older volunteers presenting higher head motion estimates (Supplementary Material B). The difference in head motion between our healthy older adults and our adults with stroke (stroke-early and stroke-late) was of no significance, allowing us to partially eliminate head motion as a driving factor of our spectral characteristic measures.

**FIGURE 1:**
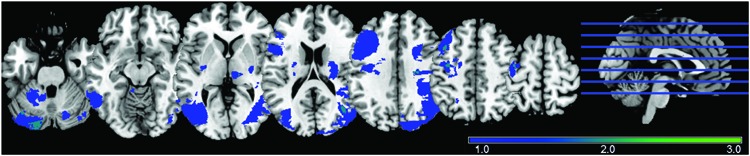
**Lesion Density Map for 30 ischemic stroke patients (early and late combined), with most lesions not overlapping**.

The stroke participants exhibited heterogenous lesions, with most exhibiting non-overlapping lesion (**Figure 1**). No lesion pertained to the main DMN oscillator of the PCC. A lesion density map, derived from semi-automated segmentation from available T1 BRAVO, Cube T2 FLAIR and Diffusion Weighted Image (DWI) using Jim 7 (Xinapse^1^), is provided describing the lesion location and lesion overlap (**Figure 1**).

### MRI Acquisition

All neuroimaging data were collected at the University of Wisconsin–Madison, using two identical 3.0-Tesla GE Discovery MR750 scanners (GE Healthcare, Waukesha, WI, USA), equipped with an 8-channel head coil. A 10-min resting state scan was acquired while the subject was instructed to lie still and relax with their eyes closed for the duration of the scan. Subjects were reminded to hold still and minimize head motion before the start of each scan. The following scanning parameters were used for the resting state scan: single-shot T2*-weighted gradient-echo echo planar imaging, with 40 sagittal slices, TR = 2.6 s, TE = 22 ms, FOV = 224 mm, flip angle = 60°, isotropic 3.5 mm × 3.5 mm × 3.5 mm voxel. The 3D high-resolution axial structural scan was acquired using a T1-weighted IR-prepared SPGR BRAVO sequence with 156 slices, isotropic 1 mm × 1 mm × 1 mm, over a 256 × 256 matrix, TR = 8.132 ms, TE = 3.18 ms, TI = 450 ms, FOV = 256 mm, flip angle = 12°. For the whole session, earplugs and foam padding were used to attenuate scanner noise and minimize head movement, respectively.

### Data Pre-processing

Pre-processing of neuroimaging data was completed using Data Processing Assistant for Resting-State fMRI (DPARSFA v2.3; Chao-Gan and Yu-Feng, 2010) and SPM8 (Wellcome Trust Centre for Neuroimaging, University of College London, UK). The first 10 volumes were discarded to allow for magnetization to reach equilibrium. The remaining images were slice-time corrected, and spatially realigned to correct for head motion. Data spikes were removed using AFNI’s *3dDespike*^2^. The image volumes were normalized to a MNI EPI template, and smoothed with a 4-mm Gaussian kernel. A group ICA on the whole set of sessions was then performed (GIFTv2.0, Calhoun, 2004), modeled with an unconstrained mid-order 28 independent component model using the *Infomax* algorithm (Bell and Sejnowski, 1995), default mask file, standard PCA type, and back-reconstruction using the GICA method. Identified components accounted for 89.1% of the total variance. Reliability of the ICA algorithm was assessed using the ICASSO toolbox^3^ (Himberg et al., 2004), with 20 iterations using *RandInit* and *Bootstrap* methods. Head motions in the participants were also computed using the Euclidean norm (enorm) method to assess the possible contribution of head motion onto results from spectral analysis.

### Analyses

**FIGURE 2:**
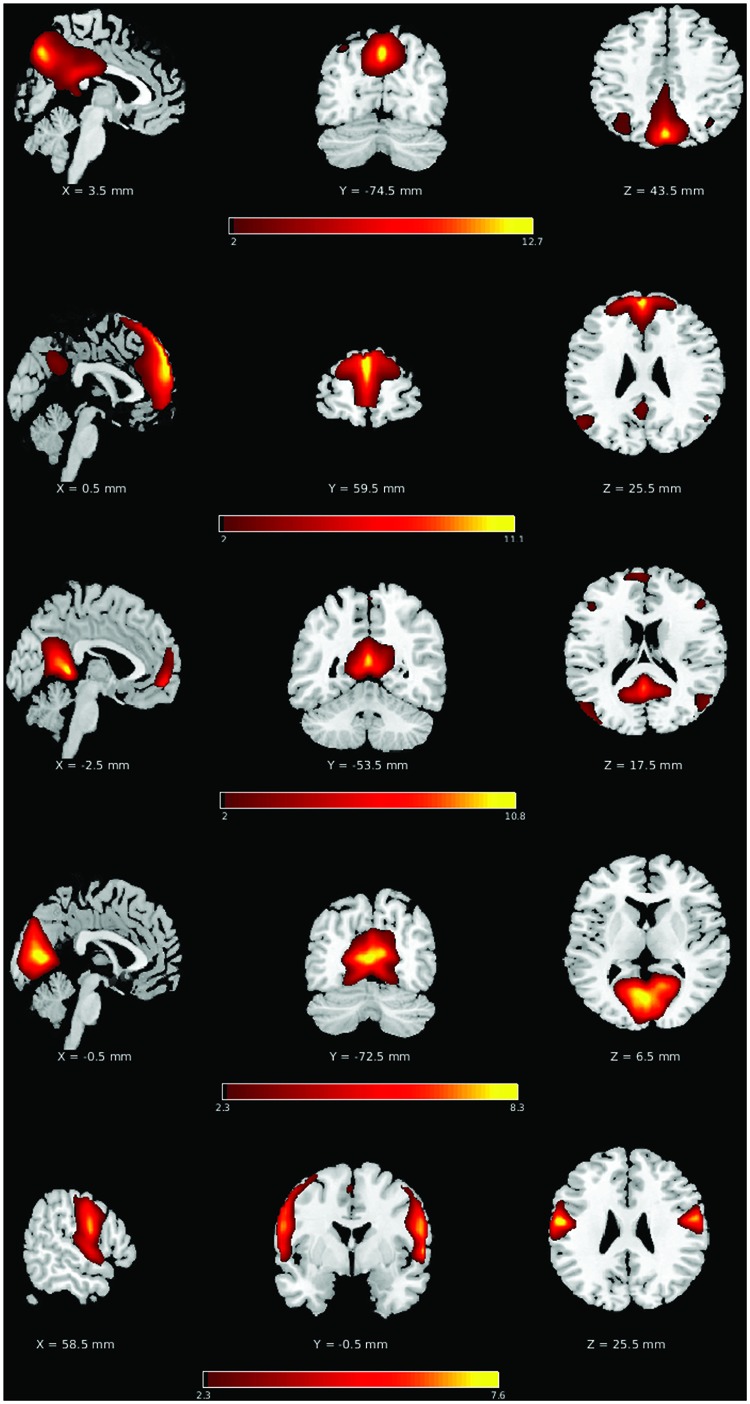
**Consistent group ICA derived components**. Top to bottom: posterior DMN (pDMN), anterior DMN (aDMN), ventral DMN (vDMN), primary visual, and primary sensorimotor, with slices chosen to allow a comprehensive depiction of the functional networks.

Twenty-one non-noise components were identified from the unconstrained groupICA, including three sub-networks of the DMN, a primary visual network component and a primary sensorimotor network component, identified by high correlation to functional network templates (**Figure 2**). The three sub-networks of the DMN consisted of a posterior DMN (pDMN), anterior DMN (aDMN), and a ventral DMN (vDMN). These five networks were selected for their robustness during the resting state scan as assessed by the *Power of Low-Frequency* (0.01–0.1 Hz) to *Power of High-Frequency* (0.1–0.192 Hz) ratio. Network robustness was defined as ratio (Power _LF_) to (Power _HF_) exceeding 50.

The components of the different sub-systems of the DMN were selected for assessment because of their high implication in healthy brain functioning as well as in advanced aging and clinical populations, but also because of their resiliency to common stroke injury from less superficial and heavily vascularized core cortical structures. The selection of this system allowed greater flexibility in the stroke lesion location, as only indirect effects (i.e., diaschisis effect) on the system is being investigated. Similarly, the early visual and sensorimotor systems were chosen for their robustness in resting state condition, as well as limited influence of stroke-induced lesion in our patient population (only one lesion in the primary visual system, and no lesion in the sensorimotor region in 32 stroke subjects).

For each investigated ICNs, a corresponding group independent component (IC) time series was obtained, followed by Fast Fourier Transform to obtain component power spectra (Calhoun et al., 2011; Gohel and Biswal, 2014). Computing measures of LFOs in the frequency domain has the advantage of offering the ability to simultaneously examine specific bands within the resting-state frequencies (0.01–0.08 Hz). To increase the consistency of the distribution across subjects, we applied smoothing with a Gaussian filter with σ = 2. The power spectra of the low-frequency oscillations were assessed with two methods: (1) a discrete bin analysis of oscillation amplitudes within the slow-5 (0.01–0.027 Hz) and some slow-4 (0.027–0.073 Hz) bands, with each bin consisting of a width of 0.0015 Hz, and (2) a measure of the fALFF in the slow-5 and in the slow-4 frequencies, both analyses implemented from the group IC time-series. The slow-5 and slow-4 oscillations ranges were identified according to the definition described by Penttonen and Buzsáki (2003) and Buzsáki and Draguhn (2004).

### Statistical Testing

Single-factor ANOVAs were used for the statistical testing of component fALFF for each of the network components, with groups [young adults (YHA), older adults (OHA), stroke-early, and stroke-late patients] serving as the single factor. This test was followed by a Tukey’s honest significant difference (HSD) *post hoc* pair-wise comparison testing between the groups. Similarly to Calhoun et al. (2011), multiple component spectra were combined to achieve better consistency in the frequency distribution of intrinsic oscillations. pDMN, aDMN, and vDMN were combined to form a representative DMN component; visual and motor were combined for a collective ‘visual–sensorimotor’ component, or ‘non-DMN’ component. Discrete frequency bin analysis of the amplitudes among the power spectra was performed using a Student’s *t*-test. That analysis was implemented from 0.009 to 0.060 Hz with a 0.0015 Hz bin increment.

## Results

Previously in a cross-sectional study with two groups of stroke patients (i.e., stroke-early and stroke-late), our group has provided evidence that a specific fluctuation range of the slow-5 oscillations (0.01–0.027 Hz) is implicated in the disruption of the DMN. More specifically the posterior component of the DMN is disrupted in the stroke-late population, with such reduction of oscillation amplitude in these frequencies potentially disrupting the communication between distal nodes within a system (La et al., 2014; La et al., submitted). Here, we examined three independent subcomponents of the DMN (pDMN, aDMN, and vDMN), as well as two components of ‘task-positive’ networks (primary visual and sensorimotor) to assess whether those findings extend beyond the DMN following the event of a stroke. We found that this reduction in slow-5 oscillations may not be exclusive to the DMN, but rather is consistent across different networks following a stroke. In contrast, healthy aging may present a different pattern of cortical disruption with some observed specificity to the DMN.

**FIGURE 3:**
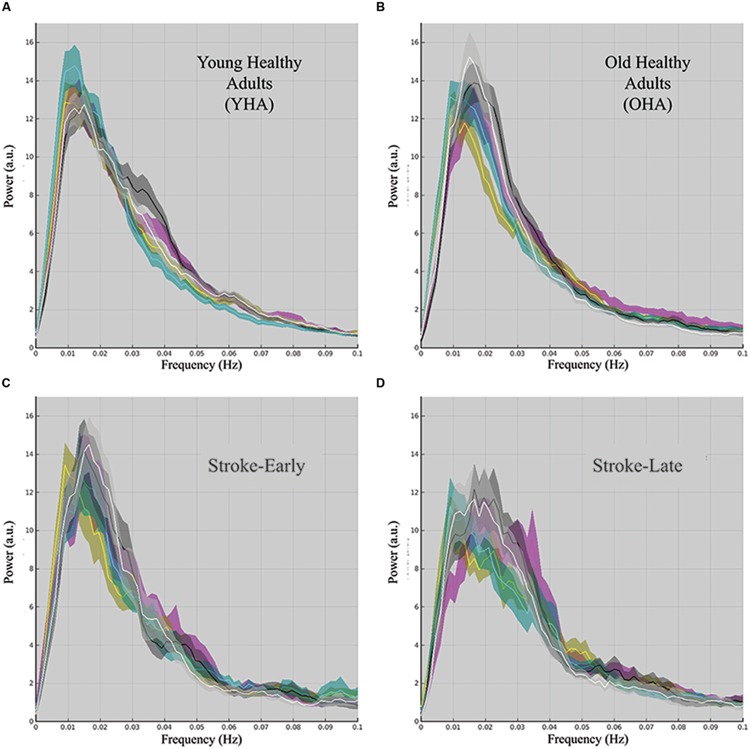
**Component spectral distribution organized by population group: (A) young healthy adult (YHA), (B) old healthy adult (OHA), (C) stroke-early, and (D) stroke-late.** Colored plots in each panel representing the mean of a distinct component, the shaded area portraying the standard error of the mean. Color coding is as follow: pDMN (magenta), aDMN (cyan), vDMN (yellow), visual (black), and motor (white). Population spectral distribution appeared highly disrupted in the stroke-late population with non-unique distribution peak, and wider spectral distribution with lower overall amplitude.

Network spectral distributions from the group IC time-series for the YHA (**Figure 3A**) and OHA (**Figure 3B**) subjects and stroke patients in their early stage (**Figure 3C**) demonstrated a stable distribution of network oscillations with a distinctive peak. Qualitatively, the stroke-early population did not present drastic differences in frequency distribution of the LFOs in comparison to the OHA participants (**Figure 3**). However, variation of the distribution among and within the different components was larger in the stroke-early group in contrast to the YHA group (**Figure 3**). These findings were also observed in the older healthy adult group (OHA), and presented no significant difference in comparison to the OHA group. Quantitatively, assessment of the measure of slow-5 component fALFF demonstrated no difference of the frequency distribution in the stroke-early population group in comparison to the OHA group (pDMN: *t* = 0.270; aDMN: *t* = 0.264; vDMN: *t* = 0.971; visual: *t* = 0.152; sensorimotor: *t* = 0.633; all with *p*-value > 0.5, **Figure 5**). The amplitudes of the frequency distribution in our stroke-early patients did not differ from that observed in the older healthy individuals. In contrast, stroke patients in their later stage (**Figure 3D**) presented a difference in the spectral distribution of the network oscillations. They exhibited a lower amplitude and wider spectral distribution common to all five assessed network components with respect to the healthy old group, at various levels of significance, potentially reflecting disrupted network oscillations within an impaired system.

**FIGURE 4:**
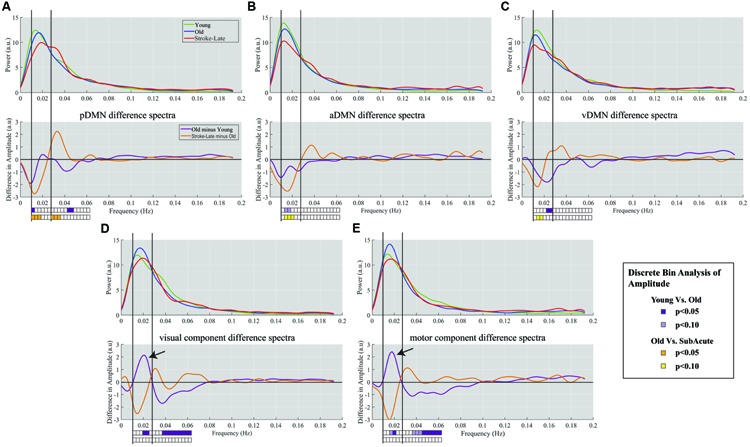
**Spectra difference plot for: Top row: each of the three components of the DMN: (A) pDMN, (B) aDMN, (C) vDMN, Bottom row: two non-DMN components of (D) primary visual, and (E) primary sensorimotor.** For each panel, top graph is a representation of the smoothed mean spectra with young (green), old (blue), and stroke-late (red). The bottom panels represent the difference between the mean spectra, with purple plot characterizing the difference between YHA and OHA, and the orange plot indicating the difference between stroke-late and OHA. Solid vertical lines demark limits of the slow-5 oscillation range (0.01–0.027 Hz) and dotted vertical lines mark the upper limit of the slow-4 frequency range (0.027–0.073 Hz). The boxes below the plots indicate the statistical significance for the discrete bin analysis of amplitude. Within the DMN, differences in stroke-late vs. OHA were noted to have large negative deflection in the slow-5 range, indicating a further reduction of the slow-5 oscillations on top of the observed reduction from aging (OHA – YHA). In the two assessed ‘task-positive’ networks, stroke-late groups exhibited a similar decrease in the oscillations. However, the aging process demonstrated a different pattern with a surge of slow-5 oscillations amplitude in those components (black arrows), coupled with a reduction of slow-4 oscillations.

An analysis of the network component oscillations established the possibility of a common pattern of disruption in the stroke-late population compared to the OHA group: a decrease in oscillation amplitude within the slow-5 oscillation range in the different components of the DMN, potentially coupled with an increase in oscillation amplitude in the slow-4 frequency, resulting in no net oscillation power increase (**Figure 4**). Discrete bin analysis with Student’s two-sample *t*-test verified the observation of a reduced oscillation amplitude in the pDMN sub-network (*p* < 0.05), and trending significance for the two DMN sub-networks (aDMN and vDMN: *p* < 0.1). Additionally, values from slow-5 fALFF were found to be lower in the stroke-late group as compared to the healthy older adult group in all five of the assessed components: from 0.383 to 0.329 in the pDMN (*t* = 1.499, *p* = 0.07, one-tailed), from 0.401 to 0.335 in the aDMN (*t* = 1.703, *p* = 0.041, one-tailed), from 0.349 to 0.305 in the vDMN (*t* = 1.623, *p* = 0.057, one-tailed), from 0.429 to 0.385 in the visual component (*t* = 1.049, *p* = 0.152, one-tailed), and from 0.437 to 0.376 in the sensorimotor (*t* = 1.274, *p* = 0.101, one-tailed). A single-factor ANOVA identified significant between-group differences in the aDMN (*F*-value = 2.815, *p* = 0.043*) and vDMN (*F*-value 3.486, *p* = 0.018*) components. *Post hoc* Tukey HSD pair-wise comparisons demonstrated significant differences in the aDMN (*p* = 0.025*) and vDMN (*p* = 0.018*) components in comparison to the YHA group, while observations in the pDMN failed to reach significance (*p* = 0.20).

**FIGURE 5:**
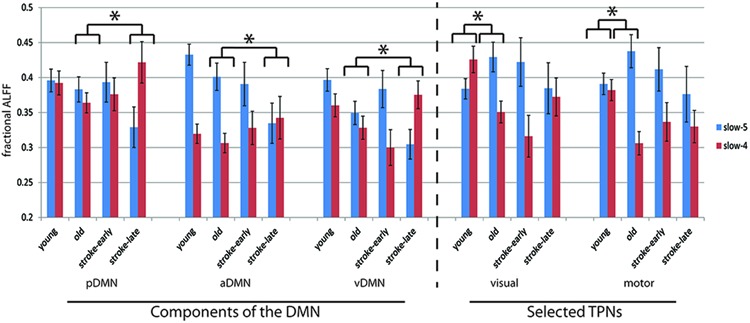
**Component fALFF for each investigated component in terms of slow-5 oscillations (0.01–0.027 Hz) and slow-4 oscillations (0.027–0.073 Hz).** In all five of the components assessed, the stroke-early group did not exhibit difference in its component fALFF measure compared to the OHA. In contrast, the stroke-late group presented a significant decrease in component fALFF in the slow-5 oscillations that was observed in pDMN, aDMN, and vDMN, while the difference observed in the visual and sensorimotor component were not significant. Aging, in the other hand, was associated with an increase in slow-5 component fALFF in those two task-positive networks (not attaining statistical significance [ANOVA], but trending in significance with two-sample *t*-test, *p* ≤ 0.1). Additionally, ratio of slow-5 to [slow-5 and slow4] combined demonstrated significance in *post hoc* two-sample *t*-tests (illustrated here by the *p < 0.05).

Despite the observed variability, these results demonstrate a consistent decrease in the slow-5 oscillation amplitude in individual DMN sub-networks. Additionally, the spectral distribution of the visual and motor components exhibited a very similar pattern of a dip in spectral distribution amplitudes within the slow-5 oscillation range (**Figures 4D,E**) as was found in the different subnetworks of the DMN. However, discrete bin (**Figure 4**, bottom) and component fALFF (**Figure 5**) analyses failed to reveal significant differences between stroke-late and the OHA groups, nor did we find significant differences between stroke-late and YHA groups, in the slow-5 range, potentially due to the limited sample size. However, a pattern of reduction of slow-5 oscillations exists. These observations suggest that a reduction in oscillations amplitude after stroke may not be specific to components of the DMN, as other networks (non-DMN networks) presented similar patterns of disruption.

**FIGURE 6:**
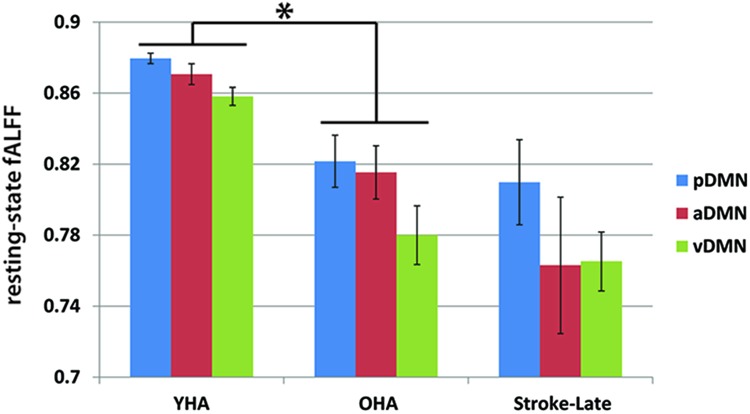
**Resting state relative oscillation power as recorded by fALFF (0.01–0.073 Hz).** Difference in resting-state fALFF between the aging-effect and the stroke-related effect. The resting-state fALFF in the OHA exhibited lower values in comparison to their younger counterpart (YHA), while levels of resting-state fALFF in the ischemic stroke-late group remained the same as the OHA, suggesting that disruption following the event of a stroke may be more due to a reallocation of the oscillation power from the slow-5 frequency range to the slow-4 frequency range, rather than a decrease of power of the total resting-state oscillations. *p < 0.05.

The lack of statistical significance of the reduction in slow-5 oscillations of task-positive networks may also be due to the difference observed in the healthy old adults with respect to the healthy young adults. Though not necessarily significant, **Figure 4** shows an inverted pattern in the two task positive networks, as part of an aging-effect (OHA compared to YHA), displaying an increase in slow-5 oscillation amplitude (ANOVA *post hoc* Tukey HSD, YHA minus OHA: visual: *p* = 0.359, sensorimotor: *p* = 0.384) with significant amplitude difference in discrete bin analysis (**Figures 4** and **5**), coupled with a significant reduction in the oscillation amplitude within slow-4 oscillations (ANOVA *post ho*c Tukey HSD, YHA minus OHA: visual: *p* = 0.016*, sensorimotor: *p* = 0.006**; **Figures 4** and **5**). Difference between the two contrasts (aging-effect versus stroke-related effect) was also present in the distribution of the resting state oscillations. The relative oscillation power estimate as recorded by fALFF over the full resting state frequencies (i.e., 0.01–0.073 Hz) to the full acquired frequency (0–0.192 Hz) remained unaltered following the onset of a stroke (pDMN: *t* = 0.273, *p* = 0.787; aDMN: *t* = 0.996, *p* = 0.331; vDMN: *t* = 0.391, *p* = 0.699), whereas resting-state fALFF exhibited lower values in the healthy older adults in comparison to the healthy younger adults (pDMN: *t* = 2.842, *p* = 0.006**; aDMN: *t* = 2.487, *p* = 0.015*; vDMN: *t* = 3.457, *p* = 0.001**; **Figure 6**).

The ratio of slow-5 oscillation power to resting-state oscillation (i.e., slow-5 plus slow-4 combined) was also affected. In the stroke-late to OHA comparison, the stroke-late group received much lower contribution of their slow-5 oscillations in the resting state (pDMN: *t* = 1.922, *p* = 0.066; aDMN: *t* = 1.791, *p* = 0.042; vDMN: *t* = 2.265, *p* = 0.015; visual: *t* = 1.075, *p* = 0.147; motor: *t* = 1.559, *p* = 0.065; *post hoc* uncorrected one-sample *t*-test; **Figure 5**). These differences in ratio were not very strong in the non-DMN components in particular, but more dramatic effects were found in the healthy older adults groups. The effects consisted of altered slow-5 to resting state oscillations ratio in the OHA in comparison to the YHA group (visual: *t* = 2.385, *p* = 0.010; motor: *t* = 2.557, *p* = 0.006; *post hoc* uncorrected one-sample *t*-test), depicting a possible age-associated change.

**FIGURE 7:**
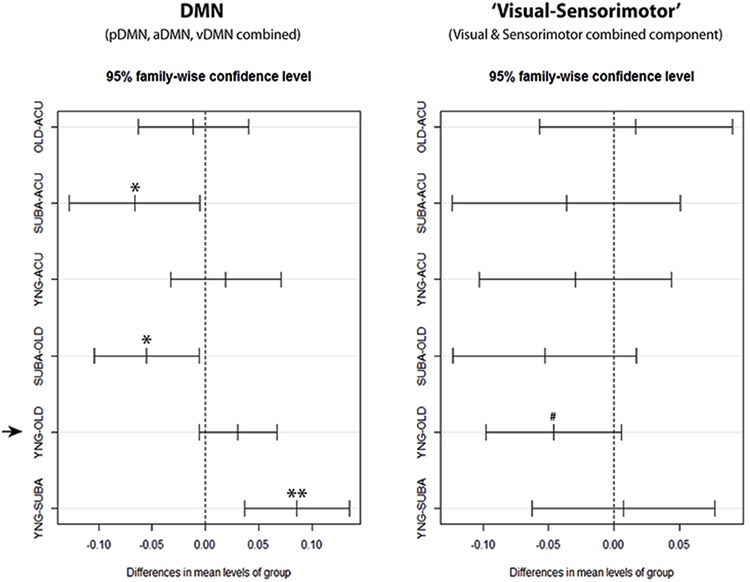
**Confidence Intervals for DMN and ‘visual–sensorimotor’ combined components single-factor ANOVA.** With the combined components, clear reduction of slow-5 fALFF in the stroke-late (SUBA) population can be observed in comparison to the acute stroke group and healthy old individuals within the DMN. Reductions in the stroke-early (ACU) group were very similar to the healthy older adults (OHA), and did not reach statistical significance. In contrast, behavior of the ‘visual–sensorimotor’ component differed between the healthy aging effect and the effect observed in the stroke-late patient population. In this ‘task-positive’ composite component, the stroke-late population exhibited a trend toward significance in a reduction of slow-5 oscillations (negative in SUBA-Old contrast), while the old presented an increase in those slow-5 oscillations compared to the young (negative in YNG-OLD contrast). **p < 0.001, *p < 0.05, ^#^*p* < 0.1.

With components combined, similarly to Calhoun et al. (2011), to form a combined-DMN component and a ‘visual–sensorimotor’ component representative of task-positive systems, single-factor ANOVA on the slow-5 component fALFF measure confirmed a difference in slow-5 disruption of cortical networks between aging and onset of an ischemic stroke. In the stroke-late group, DMN and non-DMN components showed a uniform decrease of slow-5 oscillations (Tukey HSD, stroke-late minus OHA: DMN: *p* = 0.021*, visual–sensorimotor: *p* = 0.205). In contrast, healthy older adults displayed a slight *decreased* in slow-5 oscillations, which appeared more specific to the DMN (Tukey HSD, YHA minus OHA: DMN: *p* = 0.135), as power of the slow-5 oscillations in non-DMN network *increased* (Tukey HSD, YHA minus OHA: visual–sensorimotor: *p* = 0.099; **Figure 7**). This increase in non-DMN fluctuation power and the decrease in DMN activity, both observed in the healthy older subjects, may be related.

### Brain Behavior Relationship

We have previously reported that the observed fALFF in the pDMN within the slow-5 oscillation range showed some correspondence with behavioral data (La et al., 2014; La et al., submitted). Specifically, we found significant differences in the means of the slow-5 fALFF in the pDMN between the subacute stroke patients categorized as mild (NIHSS < 2, mild stroke, *n* = 7) and subacute stroke patients categorized as moderate to severe (NIHSS ≥ 2, moderate to severe, *n* = 10) in terms of stroke severity (*t*15 = 2,488, *p* = 0.025) though the sample size were limited. Additionally, regression of out-of-scanner behavior data (phonemic verbal fluency) demonstrated a significant association between the neural measure with phonemic verbal fluency scores, with lower scores associated with lower pDMN slow-5 fALFF (*R*^2^ = 0.329, *F*15 = 7.365, *p* = 0.016). In the current study, we found no correspondence of slow-5 fALFF in these additional components (i.e., aDMN, vDMN, primary visual and sensorimotor network components) to behavior.

## Discussion

In the current study, we investigated the diaschisis effect of ischemic stroke on networks of the default-mode as well as in two ‘task-positive’ systems of the early visual and sensorimotor. By means of an analysis of group ICA derived components; our results demonstrated a possible connection between the slow-5 oscillations (0.01–0.027 Hz) and the integrity of the cortical network in terms of functional connectivity. Consistent with a previous report (Garrity et al., 2007; Zuo et al., 2010), our study demonstrated the dominance of the slow-5 oscillations in particular over regions of the DMN in the resting state frequency of young healthy volunteers (**Figure 3**), oscillations that are disrupted in the event of an ischemic stroke, altering the dominance of the slow-5 fluctuations and the balance between resting state oscillations (La et al., 2014; La et al., submitted). Furthermore, reduced slow-5 oscillation fALFF within the posterior component of the DMN (pDMN) was found to exhibit a possible association with lower behavioral scores, verifying the possible correlation between brain and behavior. In this study, we assessed whether the event of an ischemic stroke disrupts slow-5 oscillations specifically to the DMN or extends beyond this network, reflecting the state of a globally disrupted and impaired cortical system.

As the ICNs are identified in terms of the correlation of its oscillations, disturbance of such oscillations have a likelihood to interfere with the synchronization between the regions, ultimately disrupting the integrity of network functioning. The mechanism of network dis-connectivity may involve a reduction of coherence in the local neuronal firing within an oscillator or oscillator region, and/or a decrease in relevant information signaling in comparison to system noise. Regional homogeneity (Zang et al., 2004; Liu et al., 2010) offers an assessment of coherence of the local fluctuation, while localized changes in oscillation amplitudes can be investigated with an analysis of ALFF (Yu-Feng et al., 2007) and fALFF. From an assessment of network oscillation amplitudes [La et al., 2014; La et al., submitted], a method of applying fALFF approaches to ICA-derived time-series, our results presented a characterization of alterations of the cortical network in stroke populations in their early and later stages. As in our previous study [La, *submitted*], the frequency distribution of the intrinsic LFOs in the stroke-early patients did not show sign of disruption in pDMN, nor in other DMN subnetworks, primary visual and sensorimotor networks.

As shown in **Figure 3**, frequency distributions of the five assessed networks are unaltered in stroke patient in their early stage. Distributions are narrow and specific, and are comparable to distributions observed in the healthy normal groups (young and old). This may be due to an absence of diaschisis effect in the early time-point with the effect requiring more than 7-day time period of our early window to propagate to these distant networks. In contrast, the stroke population in their later stage presented a wider distribution and lower amplitude spectra in multiple network components with unchanged total oscillation power (**Figure 3D**). The negative deflection on the difference plot (**Figure 4**), reflecting of a reduction of oscillation amplitude and oscillation power within the slow-5 oscillation range, was apparent in all three of the investigated DMN subcomponents for the stroke-late population, consistent with previous findings of decreased network co-activation (Park et al., 2011; Tuladhar et al., 2013). This pattern was also observed in two ‘task-positive’ systems of the early visual and sensorimotor with differences trending toward significance when combined. The consistent reductions posit a common mechanism of network disruptions among networks of the cortical system in the stroke-late population, extending beyond the DMN. Additionally, these reductions of oscillation power suggest the impairment of slow-5 oscillation as a source of the network disruption, ultimately leading to reduced coherence within the network and the global cortical system.

Moreover, the negative deflections in the slow-5 were often paired with a positive deflection in the slow-4 oscillations (five out of the five assessed components), with no observed difference in total fluctuation power as recorded by component fALFF over the resting state frequencies (0.01–0.073 Hz). Together, this proposes a widening of the spectral distribution through a reallocation of resources with neurons potentially oscillating away from the slow-5 toward more diverse frequencies including that in the slow-4 range. This widening of distribution may contribute to a reduction of the specificity of the carried information with such diversity in oscillation frequency having a dramatic impact on the synchronization of the network, possibly impairing functions.

In the healthy older adult group, reduction of slow-5 oscillation amplitudes was also found in comparison to the YHA group to a lesser extent, but is specific to components of the DMN (**Figure 7**). This observation is consistent with previous finding of reduced DMN functional connectivity with aging (Andrews-Hanna et al., 2007; Biswal et al., 2010; Koch et al., 2010), with disruption of the slow-5 oscillation potentially contributing to the reduced DMN functional connectivity in aging. Our results also point toward a reduction of total oscillation power across the whole resting state frequency in the OHA compared to the YHA, in contrast to the stroke-late population where no difference were found in comparison to the OHA group. This supports the hypothesis of declining of resources (or reserve) within the DMN with aging as proposed by Persson et al. (2007). Regarding non-DMN systems—the visual and sensorimotor IC—both ‘task-positive’ networks—expressed an increase in slow-5 oscillation power in the OHA group (**Figure 7**) coupled with a reduction of slow-4 power, a reversal in the pattern observed in our stroke-late population. With slow-5 fluctuation as the main contributor of network dynamics, such as synchronization and power, increase in slow-5 oscillations power in those ICs may suggest a failure to properly allocate minimal resources to ‘task’-irrelevant process during the condition of rest, the resting-state. Fransson (2005) described the brain recurrently toggling between an introspectively oriented mode (‘default-mode’) and an extrospectively oriented mode, and involving attention to the external environment, balancing between independent systems. Re-allocation of resources between those independent systems is key for the normal functioning of a brain. In our aging population, proper re-allocation of the limited resources may be compromised, resulting in this elevated slow-5 oscillations in the ‘task’-irrelevant processes of visual and sensorimotor during resting condition.

Studies have also shown that aging is associated with deficit in suppression of task-irrelevant processes in working memory system (Gazzaley et al., 2005). Despite those findings being demonstrated in task paradigms, this effect may occur in resting state as well, exemplified by the elevated oscillation amplitude in the visual and sensorimotor network components. If the DMN is considered as independent network, and not necessarily having a one-to-one relationship in opposition with task-positive networks, this effect may be a consequence of a deficit in suppression of ‘task-positive’ slow-5 oscillations. Such can occur from a deficiency of the higher order cortices and their top-down modulation, or alternatively a release from DMN inhibition, directly or indirectly, ultimately increasing cortical system noise. The latter hypothesis is in concordance with the older adults having more difficulty inhibiting irrelevant information (Campbell et al., 2010; Lustig and Jantz, 2014), and our finding would suggest a possible deficit in the suppression of ‘task’-irrelevant processes during a state of resting, conceivably mirroring inhibition of task-irrelevant networks observed during task performance.

In terms of brain-behavior relationships, in a prior manuscript we found significant associations of lower slow-5 fALFF in the pDMN with verbal fluency scores, and lower slow-5 fALFF in the pDMN with higher NIH-SS scores, reflecting higher levels of impairment. In the current study, we found no association of slow-5 fALFF in the other DMN components (aDMN and vDMN), visual, and sensorimotor components with these behavioral measures. This may reflect specificity of the pDMN component and of its constituents to change in behavior, but alternatively may reflect lack of power due to our small sample size and limited behavioral variability hindering our observation of the more globalized disruption.

Despite our novel findings, the current study is not without limitations. First, our study is limited in the lack of behavioral variability. Our enrolled participants with stroke presented very limited signs of neurological deficit as seen in **Table 1**, with a within-group median of 1.5 and 2.0 in the NIH-SS. Future studies need to be done looking at wider range of behavioral scores and its association with these brain oscillations. The non-homogeneity of the stroke-induced lesions may have also contributed to a reduced efficacy of the characterization of the disrupted brain networks following a stroke. However, we still found remote or diaschisis effects with common global changes in the cortical systems regardless of the location of the stroke-induced injury. We investigated specifically three subnetworks of the DMN and two ‘task-positive’ systems, which presented limited overlap with the lesion while demonstrating robustness of their component during this state of resting, to provide a general sense of the global cortical disruption. But because of the heterogeneity and widespread distribution of these lesions, we were limited to the number of components that could be assessed. We also did not exclude patients based on severe white matter disease or previous stroke, which could also be confounding factors.

This study is also a cross-sectional investigation with each participant being scanned only once, then grouped within a population to obtain group means. The described results could have also been confounded with variability in cerebrovascular reactivity (CVR) status among the populations, and within a population as well. However, little is known regarding correction for CVR at this moment, in particular within the resting-state signal, and was therefore not directly addressed at this time, but investigations are underway. Oscillation distributions among participants are also very diverse in nature, reducing the overall significance of the findings. A study with larger sample size is needed to verify the current findings.

Additionally, measures physiologic noise, such as cardiac (Bhattacharyya and Lowe, 2004) and respiratory (Birn et al., 2006) cycles, were not specifically acquired. These are known confounding factors in the detection of low-frequency oscillations in resting-state fMRI. However, such contributions have been suggested to influence fluctuations at higher frequencies outside of the slow-5 oscillation range: 0.04 Hz for hypercapnic conditions induced by breath hold (Biswal et al., 2007), and 0.03 Hz for respiratory variations (Birn et al., 2006), and would have influenced the resting-state oscillations equally among the populations, not contributing to group differences in the oscillations amplitudes within the slow-5 range. Furthermore, ICA has been found to be relatively robust to respiration-related fluctuations, separating respiration-volume variations into separate components (Beckmann et al., 2005; Birn et al., 2008). Despite the non-dominance of slow-4 oscillations in healthy adult resting-state, this investigation could have benefitted from a more thorough exploration of the slow-4 oscillations, but because of the lack of physiological recording, a known source of noise within that frequency range, no direct claims regarding the slow-4 oscillations have been made from this investigation.

Despite the presence of other differences (e.g., vascular risk factors and/or chronic perfusion problems), our current findings suggest a strong implication of the slow-5 oscillations in network integrity, in the later stage of stroke as well as aging. However, the underlying mechanism contributing to those disruptions may differ in the two processes. Our stroke population in their later stage presented disruption of the DMN characterized by a reduction of oscillation power in the slow-5 band, coupled with a gain in power in the adjacent slow-4 band, which was also seen in task-positive networks and not unique to the components of the DMN, indicating a global impairment of cortical oscillations. Meanwhile, aging revealed a DMN-specific reduction of slow-5 with no apparent slow-4 increase, but coupled with an increase in slow-5 oscillations in non-DMN networks. This suggests aging as a more gradual process depleting cortical resources, with increase in slow-5 oscillation amplitudes in the ‘task-positive’ networks potentially related to a deficit in DMN inhibition on non-DMN networks.

## Conclusion

In stroke research, several studies have portrayed disruption of the DMN as a consequence of an onset of an ischemic stroke; however, the mechanism and the specificity of the disruption have not been described. In this study, we provided possible insights on the mechanism of the disruption by implicating the slow-5 oscillations in the integrity of the network. We have showed that disruption observed in components of the DMN following an ischemic stroke may not be specific to the DMN, but part of a more global impairment affecting the cortical system as demonstrated by the observation of similar effect in two ‘task-positive’ components of primary visual and primary sensorimotor networks. Reduction in oscillation amplitude with aging, in the other hand, may be more specific to the DMN, accompanied with increase slow-5 oscillations in the ‘task-positive’ networks. Discerning the mechanism of disruption at a more global level is critical toward the understanding of network interactions underlying brain function.

## Author Contributions

CL, RB, MM, and VP are involved in the conception and design of the study. CL, VN, and RB are involved in the development of the methods used. CL, VN, and BY are involved in the data collection. CL, PM, and VN are involved in the data analysis. CL, JS, MM, and VP are involved in the write-up of the manuscript.

## Conflict of Interest Statement

The authors declare that the research was conducted in the absence of any commercial or financial relationships that could be construed as a potential conflict of interest.

## Footnotes

1 http://www.xinapse.com/

2 http://afni.nimh.nih.gov/afni/

3 http://www.cis.hut.fi/projects/ica/icasso/
