# Supplementary material for: Differing Patterns of Altered Slow-5 Oscillations in Healthy Aging and Ischemic Stroke
Source: Front Hum Neurosci. 2016 Apr 13;10:156. doi: 10.3389/fnhum.2016.00156 (PMC4829615; doi:10.3389/fnhum.2016.00156)
Supplement: Supplementary file 1 [file Table_1.PDF]

Subject	Age (years)	Gender	Hemisphere	Lesion	Time after stroke onset (days)	Categorization	NIH-SS (0-42)
1	74	M	Bi	Occ	7	Stroke-Early	1
2	84	M	L	PeriOl	5	Stroke-Early	n/a
3	59	M	R	Front	4	Stroke-Early	2
4	79	M	L	Front	6	Stroke-Early	2
5	55	M	L	Par	3	Stroke-Early	0
6	81	F	L	Temp-Par	4	Stroke-Early	2
7	73	M	R	Ins	4	Stroke-Early	0
8	77	F	R	Cereb	1	Stroke-Early	n/a
9	57	M	L	Temp-Occ	5	Stroke-Early	0
10	45	M	L	Cereb	3	Stroke-Early	2
11	81	M	R	Thal	6	Stroke-Early	2
12	62	M	L	Ins	2	Stroke-Early	2
13	33	F	R	Cereb	7	Stroke-Early	15
14	50	M	R	Pons	7	Stroke-Early	13
15	76	F	R	Put	136	Stroke-Late	10
16	64	F	Bi	Occ	38	Stroke-Late	3
17	67	F	R	PeriOl	164	Stroke-Late	4
18	63	M	R	PeriOl	69	Stroke-Late	0
19	59	F	R	Caudate	124	Stroke-Late	2
20	57	F	L	PeriOl	175	Stroke-Late	1
21	62	M	L	Par	126	Stroke-Late	0
22	75	M	L	Occ	146	Stroke-Late	1
23	41	F	L	Ins	31	Stroke-Late	n/a
24	65	M	R	Thal	165	Stroke-Late	0
25	49	M	R	Temp-Par	122	Stroke-Late	6
26	71	M	R	IntCap	24	Stroke-Late	2
27	69	M	L	Cereb	74	Stroke-Late	0
28	52	F	L	Cereb	180	Stroke-Late	1
29	45	F	R	Cereb	31	Stroke-Late	0
30	53	M	R	Cereb	155	Stroke-Late	0

Supplemental Material A. Clinical and Demographic Information for the 30 Ischemic Stroke patients enrolled (Stroke-Early and Stroke-Late). Note: Occ, Occipital; PeriOl, PeriOlandic; Front, Frontal, Temp-Par, Temporo-Parietal; Front-Par, Front-Parietal; Par, Parietal; Ins, Insula, Cereb, Cerebellum; Temp-Occ, Temporal-Occipital; Ins, Insula; Put, Putamen; Caud, Caudate; IntCap, Internal Capsule. * denotes patients with DMN lesion who were excluded from the study analysis.
