# Supplementary material for: Differing Patterns of Altered Slow-5 Oscillations in Healthy Aging and Ischemic Stroke
Source: Front Hum Neurosci. 2016 Apr 13;10:156. doi: 10.3389/fnhum.2016.00156 (PMC4829615; doi:10.3389/fnhum.2016.00156)
Supplement: Supplementary file 2 [file Image_1.PDF]

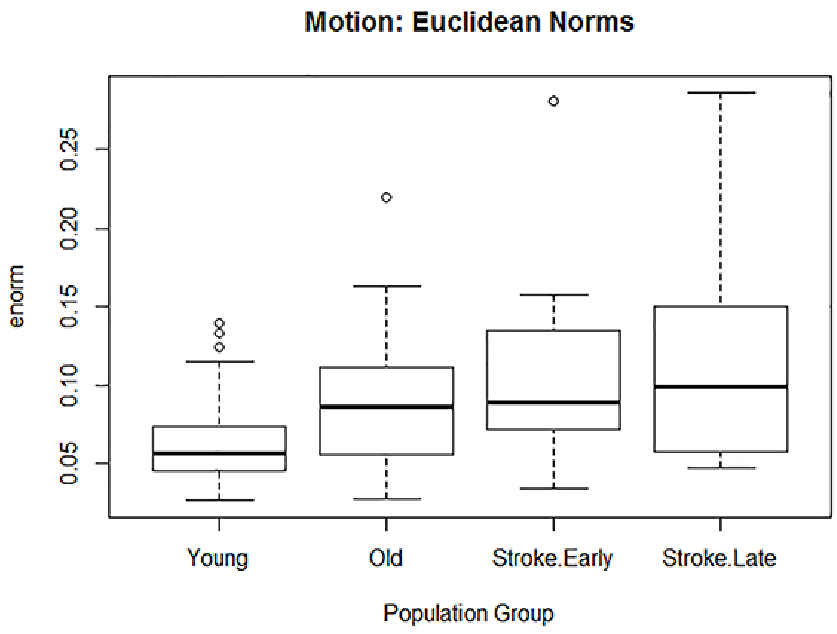

Supplemental Material B. Euclidean Norms for Population group motion estimates. Motion estimate by Euclidean norm per population groups with age demonstrated as a factor for head motion (i.e. older subjects with higher head motion) (Young vs. Old: t=3.145, p=0.001**), but with head motion in the stroke-early and stroke-late groups not differing from motion observed in the healthy older group (Old vs. Acute: t=1.134, p=0.273; Old vs. Subacute: t=1.643, p=0.117)
